# The complete chloroplast genome of the Egyptian henbane (*Hyoscyamus muticus* L., Solanaceae)

**DOI:** 10.1080/23802359.2022.2086493

**Published:** 2022-06-30

**Authors:** Mahmoud Magdy, Esraa Attia El-Sherbeny, Agripina Ramirez Sanchez

**Affiliations:** a Department of Genetics, Faculty of Agriculture, Ain Shams University, Cairo, Egypt; b Department of Genetics Resources, Desert Research Center, Ministry of Agriculture, Cairo, Egypt; c Faculty of Education Science, Santo Domino Autonomous University, Santo Domingo, Dominican Republic

**Keywords:** *Hyoscyamus muticus*, Egyptian henbane, chloroplast genome, NGS, phylogenetic analysis, inverted repeats

## Abstract

Egyptian henbane (*Hyoscyamus muticus* L. Mant. Pl. 1767) is an important medicinal plant of the family Solanaceae. Its complete chloroplast (cp) genome was assembled using Illumina high-throughput sequencing technology to contribute to its conservation genetics studies. Here, we report the complete sequence of the chloroplast genome of *H. muticus*. The cp genome was 156,271 bp in length with an asymmetric base composition (30.9% A, 18.9% C, 18.6% G and 31.6% T). It encodes 132 genes comprising 87 protein-coding genes, 29 tRNA genes, and 8 rRNA genes. The overall GC content of the *H. muticus* chloroplast genome was 37.5%, and the corresponding values in the large single-copy (LSC), the small single-copy (SSC), and the inverted repeat (IR) regions were 35.5%, 31.5%, and 43.0%, respectively. The complete chloroplast genome sequence was deposited to the GenBank (NCBI, Accession number: MZ450974). The maximum likelihood phylogenetic analysis showed that the *H. muticus* and *H. niger* were clustered into one clade with strong support values, indicating their closer relationship.

Egyptian henbane (*Hyoscyamus muticus*) is an endemic solanaceous plant in Egypt and one of the most important medicinal herbs produced in Egypt, which dates back to ancient history (Mahran 1967; Sevon et al. 2001). At least 11 species of the genus Hyoscyamus were recorded from Asia, North Africa, Europe and reaching out to Canary Islands in the Atlantic Ocean. *Hyoscyamus niger*, *H. albus*, and *H. muticus* were the most studied species, where all are pharmaceutically similar and important (Sevon et al. 2001). They are famous natural sources of the tropane alkaloids (e.g., hyoscyamine, scopolamine, and atropine) and are known for their medicinal, hallucinogenic, and poisonous properties (Xiao and He 1983; Tetenyi 1987). These alkaloids are parasympathomimetic substances that mimicking or modifying the effects of acetylcholine by the stimulation of the central nervous system (Roddick 1991). However, the chloroplast genome of *H. muticus* has yet to be published. In this study, we reported and characterized the complete chloroplast genome of *H. muticus* based on the next-generation sequencing method.

The fresh leaves were collected from *H. muticus* plants found on small inner islands in the Nasser Lake in Abu Simbel city of southern Egypt (22°29′23.2″N 31°47′44.3″E) according to the ethical guidelines of Ain Shams University (Cairo, Egypt) and approved through the administrative legalization channels of Desert Research Center head office (Cairo, Egypt). Seeds from the sampled *H. muticus* plants were stored in the applied genomics and biodiversity laboratory plant collections at Ain Shams University (voucher ID: LX00232; managed by the corresponding author). According to the manufacturer's manual, total genomic DNA was extracted using Wizard® Genomic DNA Purification Kit (PROMEGA, Madison, WI). The whole-genome sequencing was conducted on the Illumina HiSeq 2000 (Novogene, Beijing, China) with ∼300 bp insert size at 11× sequence depth. Clean pair-end reads were filtered, and *de novo* assembled using the single-contig approach (Magdy et al. 2019; Magdy and Ouyang 2020). The complete chloroplast genome was annotated as a circular molecule using the online annotation tool GeSeq (Tillich et al. 2017), whereas tRNAscan-SE V2 was used to find and annotate tRNA genes. All the annotated CDSs were verified and corrected by translation using Geneious R10 (Kearse et al. 2012). Finally, the genome map was generated by using the webserver OGDRAW (https://chlorobox.mpimp-golm.mpg.de/OGDraw.html).

The chloroplast genome of *H. muticus* is a double-stranded, circular DNA molecule and had a quadripartite structure similar to most land plant chloroplast genomes. The whole cp genome of *H. muticus* is 156,271 bp in length, containing 123 genes and 119 intergenic spacers (IGS) regions. Out of the 123 genes, 87 are coding sequences (CDS), 29 tRNA genes, and 8 rRNA genes. In detail, it is composed of two inverted repeats (IRA and IRB), both of which were 51,572 bp, separated by a large single-copy region (LSC, 86,764 bp) and a small single-copy region (SSC, 17,935 bp). The recorded GC content was 37.5%. All genes were single-copy except for 16 genes were double-copies. The double-copy genes were six protein-coding genes (*rpl*2, *rpl*23, *ycf*2, *ndh*B, *rps*7, and *ycf*1), four rRNAs (rRNA 5S, rRNA 4.5S, rRNA 23S and rRNA 16S) and five tRNAs (*trn*L^CAA^, *trn*N^CUU^, *trn*R^ACG^, *trn*V^GAC^, and *trn*M ^CAU^) and were located in the IR. Among annotated genes, eight genes (*atp*F, *ndh*A, *ndh*B, *rpo*C1, *pet*D, *pet*B, *rpl*16, and *rpl*2) harbored one intron, and three genes (*clp*P1, *rps*12, and *ycf*3) harbored two introns.

Phylogenomic analysis was performed using complete chloroplast genomes of *H. muticus* and other six species from the Solanaceae family along with the *Hordeum jubatum* (Foxtail Barley) as an outgroup. All chloroplast genomes were compared using Mauve aligner (Darling et al. 2004) and the tree was computed using FastTree V2 (Price et al. 2010). The maximum likelihood-based phylogeny revealed the kinship of *H. muticus* and *H. niger* with the highest bootstrap support values (1.00) compared to other genera of the family Solanaceae (Figure 1). The sequenced chloroplast genome was confirmed as a member of the genus Hyoscyamus and is the first completed chloroplast genome of *H. muticus* to be announced. The chloroplast genome should provide informative data both for the conservation genetics and any future plastomic and/or evolutionary studies of the genus Hyoscyamus and family Solanaceae. A family that has significantly contributed to molecular plant genetics and genomics since the beginning of the twentieth century (Gebhardt 2016).

**Figure 1.**
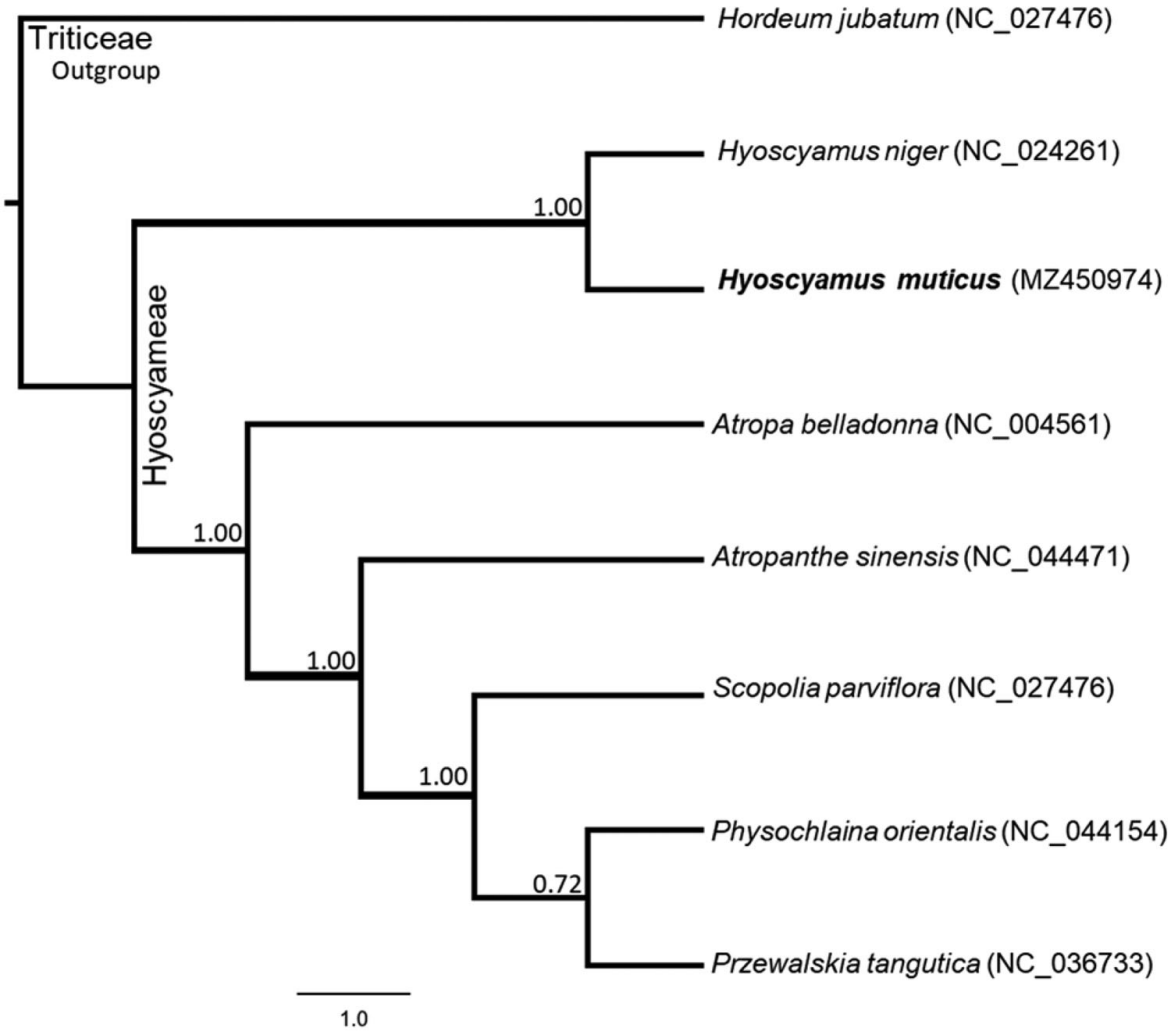
Maximum-likelihood tree based on eight complete chloroplast genomes. The number on each node indicates the bootstrap value (at 1000 runs). *Hyoscyamus muticus* is shown in bold, and GenBank accession numbers are listed for each species name.

## Data Availability

The genome sequence data that support the findings of this study are openly available in GenBank of NCBI at https://www.ncbi.nlm.nih.gov/ under the accession no. MZ450974. The raw data was submitted as fastq files to the SRA data no. SRR17602593, and the samples were registered as BioSample no. SAMN24920067 and BioProject no. PRJNA796812.
